# Intraoperative Near-Infrared Fluorescence Imaging with Indocyanine Green for Identification of Gastrointestinal Stromal Tumors (GISTs), a Feasibility Study

**DOI:** 10.3390/cancers14061572

**Published:** 2022-03-18

**Authors:** Gijsbert M. Kalisvaart, Ruben P. J. Meijer, Okker D. Bijlstra, Hidde A. Galema, Wobbe O. de Steur, Henk H. Hartgrink, Cornelis Verhoef, Lioe-Fee de Geus-Oei, Dirk J. Grünhagen, Yvonne M. Schrage, Alexander L. Vahrmeijer, Jos A. van der Hage

**Affiliations:** 1 Department of Surgical Oncology, Leiden University Medical Center, 2333 ZA Leiden, The Netherlands; r.p.j.meijer@lumc.nl (R.P.J.M.); o.d.bijlstra@lumc.nl (O.D.B.); w.o.de_steur@lumc.nl (W.O.d.S.); h.h.hartgrink@lumc.nl (H.H.H.); y.schrage@nki.nl (Y.M.S.); a.l.vahrmeijer@lumc.nl (A.L.V.); j.a.van_der_hage@lumc.nl (J.A.v.d.H.); 2 Department of Radiology, Leiden University Medical Center, 2333 ZA Leiden, The Netherlands; l.f.de_geus-oei@lumc.nl; 3 Department of Surgical Oncology and Gastrointestinal Surgery, Erasmus MC Cancer Institute, 3015 GD Rotterdam, The Netherlands; h.galema@erasmusmc.nl (H.A.G.); c.verhoef@erasmusmc.nl (C.V.); d.grunhagen@erasmusmc.nl (D.J.G.); 4 Department of Surgical Oncology, Antoni van Leeuwenhoek, The Netherlands Cancer Institute, 1066 CX Amsterdam, The Netherlands

**Keywords:** gastrointestinal stromal tumor (GIST), near-infrared fluorescence imaging, image-guided surgery, indocyanine green (ICG)

## Abstract

**Simple Summary:**

Surgical resection plays a pivotal role in the treatment of GIST patients. The current study aims to explore the use of near-infrared fluorescence imaging to optimize the intraoperative tumor identification of GISTs. For this purpose, the potential and limitations of the widely used, and non-specific, tracer indocyanine green were assessed in a multicenter study including 10 patients. Our results show that GISTs typically have similar fluorescence intensity to the surrounding tissue, within several minutes after the intravenous administration of indocyanine green. These findings justify future research into specific fluorescent tracers for GISTs, and set a reference for future intraoperative imaging trials.

**Abstract:**

Background: Optimal intraoperative tumor identification of gastrointestinal stromal tumors (GISTs) is important for the quality of surgical resections. This study aims to assess the potential of near-infrared fluorescence (NIRF) imaging with indocyanine green (ICG) to improve intraoperative tumor identification. Methods: Ten GIST patients, planned to undergo resection, were included. During surgery, 10 mg of ICG was intravenously administered, and NIRF imaging was performed at 5, 10, and 15 min after the injection. The tumor fluorescence intensity was visually assessed, and tumor-to-background ratios (TBRs) were calculated for exophytic lesions. Results: Eleven GIST lesions were imaged. The fluorescence intensity of the tumor was visually synchronous and similar to the background in five lesions. In one lesion, the tumor fluorescence was more intense than in the surrounding tissue. Almost no fluorescence was observed in both the tumor and healthy peritoneal tissue in two patients with GIST lesions adjacent to the liver. In three GISTs without exophytic growth, no fluorescence of the tumor was observed. The median TBRs at 5, 10, and 15 min were 1.0 (0.4–1.2), 1.0 (0.5–1.9), and 0.9 (0.7–1.2), respectively. Conclusion: GISTs typically show similar fluorescence intensity to the surrounding tissue in NIRF imaging after intraoperative ICG administration. Therefore, intraoperatively administered ICG is currently not applicable for adequate tumor identification, and further research should focus on the development of tumor-specific fluorescent tracers for GISTs.

## 1. Introduction

Gastrointestinal stromal tumors (GISTs) are mesenchymal tumors characterized by their differentiation towards the interstitial cells of Cajal [1]. GISTs mainly originate in the stomach and small bowel (approximately 60% and 30%, respectively), but can be found anywhere in the gastrointestinal tract [2]. Surgical resection is the standard treatment in localized GISTs. Furthermore, neoadjuvant imatinib treatment might be effective in GISTs with tyrosine kinase inhibitor (TKI)-sensitive mutations if a reduction in tumor volume is expected to enable less mutilating surgery. In advanced GISTs, the role of surgery is more complicated. Systemic treatment is the standard of care for these patients. However, in retrospective studies, surgery has been associated with prolonged survival in patients with (oligo)metastatic disease that is responsive to imatinib [3,4,5,6]. The causality remains unclear in this association. Nevertheless, after thorough consideration of the implications of surgery on an individual patient level, surgical resection might be indicated for specific advanced GIST patients [7].

In localized and advanced GISTs, accurate intraoperative tumor identification is of great importance for achieving successful resections. Both tumor-positive resection margins and tumor rupture during surgery are linked to an impaired prognosis [8,9]. In GISTs specifically, intraoperative tumor identification can be complicated by intraluminal growth and minimal tactile feedback on palpation, due to soft and elastic tumor tissue. In other malignancies, near-infrared fluorescence (NIRF) imaging, with both tumor-specific and non-specific fluorescent tracers, such as indocyanine green (ICG) and methylene blue, has been successfully used to assist surgeons with the identification and demarcation of tumor tissue and surrounding vital structures [10,11,12,13]. Tumor identification with NIRF imaging, after the intraoperative administration of ICG, has been reported to be feasible, due to the biological mechanisms of perfusion, vessel permeability, and retention in multiple non-hepatic abdominal tumors [10,14]. In GISTs, however, the application of NIRF imaging is still unexplored. The current study primarily aims to determine the potential and feasibility of NIRF imaging for the intraoperative identification of GISTs, with the widely used, non-specific, fluorescent tracer ICG, after intraoperative intravenous administration. The secondary aim is to semi-quantify GIST fluorescence after ICG administration with tumor-to-background ratios (TBRs), improving the interpretability of the result and allowing for comparisons with future studies.

## 2. Materials and Methods

### 2.1. Design and Patients

The study protocol was approved by the Medical Ethics Committee Leiden Den Haag Delft (METC LDD) and registered at ClinicalTrials.gov with the identifier NCT04761172. The study was designed as a multicenter open-label pilot study, aiming to include a total of 10 patients to determine feasibility. Patients were included and treated between May 2020 and March 2021 in the Leiden University Medical Center (LUMC) and the Erasmus Medical Center Cancer Institute (EMC), which are both referral centers for GIST patients. Histologically or cytologically proven GIST patients, who were scheduled for open or laparoscopic resection, were screened for inclusion. The exclusion criteria were an age younger than 18 years and contraindications for ICG, i.e., pregnancy, severe renal insufficiency (eGFR <30 mL/min/1.73 m^2^), or an allergy to iodine or ICG. Furthermore, patients with esophageal GIST were excluded, since these GISTs were anticipated to be inadequately approachable with the NIRF camera. All included patients gave written informed consent.

### 2.2. Near-Infrared Fluorescence Imaging

Directly before the start of surgery, a 10 mg ICG (2.5 mg/mL) solution in sterile water was prepared (Verdye^®^ Diagnostic Green GmbH, Asscheim, Germany). After opening of the abdominal cavity and visual inspection of the tumor location, the ICG solution was intravenously administered as a single bolus. Intraoperative NIRF imaging of the GIST lesions was performed with the Quest Spectrum system (Model 2.0. Quest, Middenmeer, The Netherlands). Guided by the methods used in studies on the use of ICG and intraoperative NIRF imaging for the identification of peritoneal non-GIST lesions, static NIRF images were acquired at 5, 10, and 15 min after the ICG injection [10,11,12,13,15,16].

### 2.3. Analysis

The fluorescence of GISTs and the surrounding tissue was qualitatively evaluated during surgery. For quantitative comparison, tumor-to-background ratios (TBRs) were calculated for identified GIST lesions at every imaging timepoint. TBRs were calculated as TBR = µ𝐼t/µ𝐼b, where µ𝐼t is the mean pixel intensity of the tumor tissue on the NIRF image, and µ𝐼b is the mean pixel intensity of the background, i.e., an approximately 1 cm broad ring of healthy tissue directly surrounding the tumor. Both the tumor and the background areas were manually drawn on a white-light image, and registered on the corresponding NIRF image with ImageJ (v1.53. Rasband, W.S., ImageJ, U.S. National Institutes of Health, Bethesda, MD, USA). Due to the intense fluorescence of liver tissue after ICG injection, liver tissue was excluded from the annotated background areas. TBRs were not calculated for non-exophytic GISTs, since the adequate identification of tumor tissue on intraoperative white-light images is a requisite for accurate annotation. For result analysis, Python was used (v3.9. Python Software Foundation. Available at http://www.python.org, accessed on 3 December 2021).

## 3. Results

### 3.1. Patient and Tumor Characteristics

A total of 10 patients were treated in this study, of which 7 were in LUMC and 3 were in EMC (Table 1). A total of 11 GIST lesions were resected. In nine patients, the resection of a primary localized GIST was performed, while one patient was treated with cytoreductive resection of two known GIST lesions. In seven patients, the GIST was located in the stomach, while, in two patients, the lesions originated from the ileum, and in one patient, the lesion originated from the duodenum. The lesion diameters on preoperative CT ranged from 25 to 150 mm, with a median of 50 mm. Exophytic growth was observed in eight lesions, intraluminal growth was observed in two lesions, and one GIST was located submucosally. The number of mitoses per 5 mm^2^ was determined on biopsy material acquired before treatment in eight patients, and ranged from 2 to 47, with a median of seven. Furthermore, in eight patients, a KIT exon 11 mutation was found, and in one patient, a PDGFRA exon 18 mutation was detected. No information on the mutation was available for one patient, due to a diagnostic workup in a referring center. Half of the patients were treated with TKIs before surgery. Depending on the tumor size and location, four patients underwent an open procedure and six patients underwent laparoscopic procedures. Pathologic evaluation of the resected lesions confirmed the diagnosis of GISTs in all cases. Moreover, in the five patients who underwent neoadjuvant treatment, the percentage of remaining viable GIST cells ranged from 0% to 100%, with a median of 75%.

### 3.2. Qualitative Analysis

**Figure 1:**
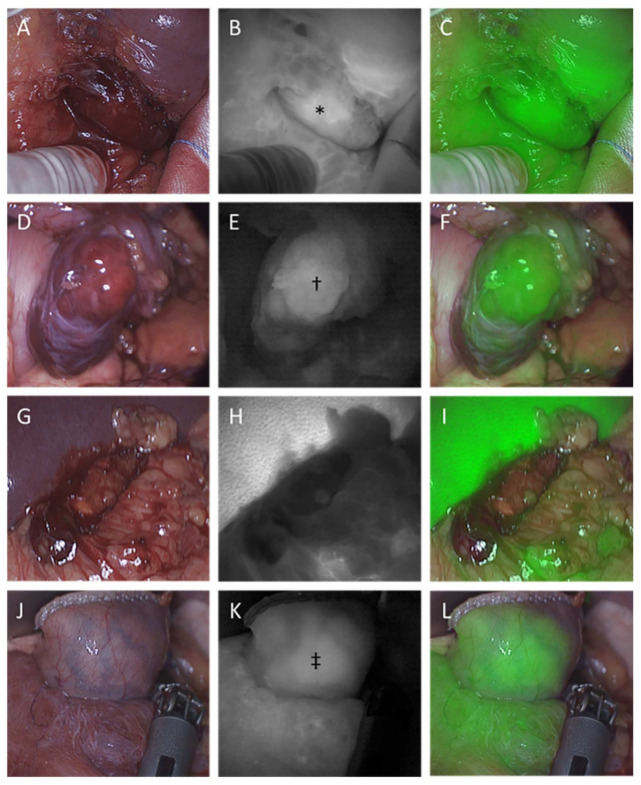
Imaging of 4 cases representing examples of all 4 scenarios described in the qualitative analysis results section, at 10 min after ICG administration. (**A**,**D**,**G**,**J**) Intraoperative white-light images. (**B**,**E**,**H**,**K**) Corresponding NIRF images. (**C**,**F**,**I**,**L**) Corresponding fused images. (**A**–**C**) Patient 6. An exophytic GIST arising from the duodenum, after imatinib treatment. The fluorescence in the lesion is similar to that in the surrounding tissue. * A hyperfluorescent region within the diffusely fluorescent tumor, endorsing the biological tumor heterogeneity, possibly caused by the preoperative systemic treatment. The resected tumor was found to have 50% of tumor cells remaining. (**D**–**F**) Patient 1. An exophytic GIST arising from the stomach during laparoscopy, showing relatively high and specific fluorescence, and the highest TBR in the current study. ^†^ In particular, the central tumor bulk shows intense fluorescence. (G–I) Patient 8. An exophytic GIST arising from the stomach, showing close to no fluorescence, while intense fluorescence is observed in the adjacent liver tissue. The lowest TBRs were calculated in this GIST for every timepoint. (J–L) Patient 2. An intraluminal GIST of the stomach. The stomach shows diffuse fluorescence. ^‡^ A region of the stomach wall, at the tumor location, shows more intense fluorescence than the surrounding stomach tissue. Nevertheless, directly after resection, no specific tumor fluorescence could be observed in the resected material.

NIRF imaging was performed in all 11 GIST lesions. In general, synchronous fluorescence of the GIST tissue and surrounding tissue was observed within several minutes (Figure 1). At 15 min after ICG administration, synchronous wash-out of ICG was typically observed in both the tumor and healthy tissue. In five lesions, including the two recurrent lesions in patient number 5, the fluorescence intensity was visually similar for the GIST and the surrounding tissue, independent of the timing of imaging. Furthermore, in one exophytic gastric lesion with a PDGFRA mutation, the fluorescence intensity was found to be relatively specific for the tumor tissue, in the qualitative assessment. In contrast, partly due to overexposure with NIRF light radiating from the adjacent liver tissue, nearly no fluorescence in the GISTs and surrounding peritoneal tissue was detected in two patients with stomach GISTs. Moreover, in three non-exophytic GISTs, the tumor tissue could not be directly visualized in white light, nor identified with NIRF imaging, intraoperatively.

### 3.3. Semi-Quantitative Analysis

**Figure 2:**
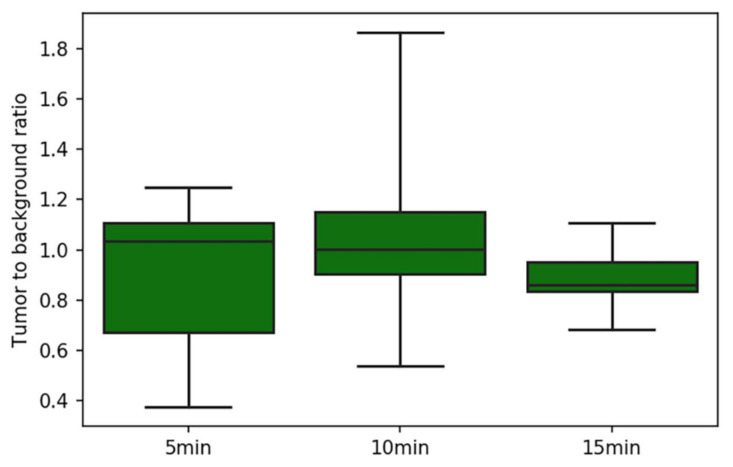
TBRs measured at 3 timepoints. Boxplots represent the median, quartiles, and range of the population.

As stated, in three non-exophytic GIST lesions, no intraoperative TBR was calculated. Furthermore, due to a technical error, no static images were available for analysis in patient number 4. As a result, seven lesions were semi-quantitatively analyzed at separate timepoints after ICG administration (Figure 2). The median TBR for all the lesions at all the timepoints was 1.0, and all the TBRs ranged from 0.4 to 1.9. The median TBRs were 1.0 at 5 and 10 min after ICG administration, and 0.9 at 15 min after ICG administration. The corresponding ranges were 0.4–1.2, 0.5–1.9, and 0.7–1.2 for 5, 10, and 15 min after ICG administration, respectively. The highest TBR was found in patient number 1, which corresponds to the qualitative intraoperative evaluation observed in this patient. The two lesions in which nearly no fluorescence was observed, in both the GIST and surrounding tissue, had the lowest TBRs.

## 4. Discussion

Intraoperative tumor identification is pivotal for the adequate surgical treatment of GISTs. In this regard, it is hypothesized that intraoperative NIRF imaging might improve the ability of surgeons to localize and demarcate tumor tissue in GIST patients. Overall, the results of this pilot study show the fluorescence intensity, within minutes after ICG administration, to be similar in GISTs as in the surrounding peritoneal tissue. Therefore, intraoperatively administered ICG is not feasible for adequate intraoperative tumor identification.

To the best of our knowledge, this study is the first clinical study on the use of NIRF imaging in GISTs. Two case reports describe the use of intraoperative NIRF imaging in GIST patients. Although the comparability of the results is limited, our findings are in contrast with a case report describing two laparoscopic partial gastrectomies for GISTs. In these patients, quick fluorescence of the tumor tissue after ICG injection was observed [17]. At least one of these two lesions is reported to have an intraluminal growth pattern. Although the same amount of ICG was administered as in the current study, the timing of imaging, TBRs, and other specifications are not reported, complicating the interpretation of these results. The second case report describes the absence of fluorescence in GIST metastasis, localized in liver tissue 24 h after ICG injection [18]. The underlying biological mechanism causing the contrast in tumor and liver fluorescence is the fast clearance and accumulation of ICG in the healthy hepatic tissue surrounding the GIST metastasis. In the current study, no hepatic GIST lesions are included, and the underlying mechanisms for ICG distribution are based on perfusion, permeability, and retention.

In non-GIST malignancies, tumor lesions with comparable anatomical tumor location and growth are reported to show fluorescence after intraoperative ICG administration. For example, several studies show that peritoneal metastasis in ovarian and colorectal cancer can be detected with NIRF imaging, within 5–50 min after the ICG injection [10,11,12,13,15,16]. Although conclusions about the usefulness vary, these studies strike a consensus regarding the specificity of the fluorescence intensity of tumor tissue, in comparison to surrounding peritoneal tissue. The reported TBRs typically range from 1.3 to 2.0, indicating that ICG is more specific for those types of malign lesions than the exophytic GISTs in the current study.

Interestingly, Liberale et al. showed an ability to differentiate between mucinous and non-mucinous colorectal cancer using ICG [10]. The mean TBR in the 21 mucinous lesions was 0.98, while the non-mucinous lesions had a mean TBR of 1.92. In GISTs, intertumoral biological heterogeneity might also partially cause variability in tumor fluorescence, as found in the current study. Of note is the presence of the only PDGFRA exon 18 mutation in the one lesion displaying GIST-specific fluorescence, while all the KIT exon 11 mutation-related GISTs showed no specific fluorescence. Furthermore, half of the patients in the current study were treated with TKIs before surgery, and the remaining number of viable cells varied widely within this subgroup. Hence, the fluorescence due to ICG might vary for subgroups of GISTs with certain characteristics, which can only be identified in larger cohorts.

Two recent studies evaluated the use of ICG and intraoperative NIRF imaging in other malignancies of mesenchymal origin. A total of 39 patients with high-grade sarcoma, of which 28 were soft-tissue sarcoma (STS), were included in these studies [19,20]. In contrast with the intraoperative ICG administration used in the current study, ICG was typically administered 16 to 24 h before surgery. Fluorescence was reported in 26 STS. The interpretability and comparability of this result is somewhat limited, since quantitative analysis is lacking and the reported NIRF images present heterogeneous tumor fluorescence of unclear specificity. However, considering these results, it is conceivable that the preoperative administration of ICG would improve the usefulness of NIR imaging in GISTs, due to the enhanced permeability and retention, or cell uptake, of ICG in the GIST tissue. In multiple murine models with other tumor types, TBRs were reported to rise within minutes after the injection, reaching a plateau that remained for several days [21,22]. However, previous studies that administered ICG 24 h prior to the resection of non-hepatic abdominal tumors generally found no fluorescence signal during surgery [10,11,12,13,15,16]. In the studies concerning sarcomas, a relatively low tumor grade has also been linked to a lower rate of ICG uptake in tumor cells, possibly limiting the usefulness of preoperatively administered ICG in GISTs [20,23]. Nevertheless, future studies regarding this topic should consider the differences in distribution mechanisms after intraoperative or preoperative ICG injection, and compare the use of ICG administered 24 h prior to surgery to the current results.

The mechanisms of perfusion, permeability, retention, and cell uptake underlying ICG distribution are not specific for tumor tissue. However, if they are substantially enhanced in the tumor tissue, in contrast to the healthy surrounding tissue, NIRF imaging with ICG can visualize this difference. The results of the current study show that these mechanisms do not allow for visual differentiation between the GIST and surrounding tissue, within 15 min after ICG injection. Since all the mechanisms underlying ICG distribution are non-specific for tumor cells, it has been suggested, in the literature, that ICG is inherently insufficient for optimal tumor tissue identification [24]. Visual differentiation on NIRF imaging might be more feasible with a fluorescent tracer that specifically binds tumor cells. Approximately 95% of GISTs have strong and diffuse overexpression of tyrosine kinase KIT [25,26]. Indeed, several cell line and mural studies have shown the potential of fluorescence-labeled anti-KIT antibodies [27,28,29]. Next to KIT, the expression of DOG1 is observed in >95% of GISTs [26]. These immunophenotypic characteristics of GISTs provide possible opportunities to develop fluorescent tracers that bind GIST cell-specific biomarkers and improve the feasibility of intraoperative NIRF imaging. Future research should aim to explore the potential of tracers that bind KIT, DOG1, and other biomarkers, described in the literature as helpful for NIRF imaging in malignancies with similar immunophenotypic characteristics to GIST [30].

A strength of the current study is the heterogeneity of the included GISTs, allowing a broad assessment of the use of NIRF imaging with ICG in patients with different characteristics. The multicentric design of this study partly assents that the results reported are repeatable, and the negative results are not likely to be refuted in future studies with comparable methods. Furthermore, since future studies testing specific GIST tracers are anticipated, this study provides a quantitative reference point in the use of a relatively cheap, non-specific tracer, on which new results should be measured.

A limitation of our study is the limited number of patients included, prohibiting robust conclusions in specific subgroups of the GIST population. Nevertheless, the aim of the study was to provide a conception of the feasibility of the use of intraoperatively administered ICG. Moreover, the use of NIRF microscopy imaging of the resected material would have provided a more profound insight into the specificity of GIST tissue fluorescence. The pathological validation of tracer distribution might also bring the potential reasons for heterogenous intra- and intertumoral fluorescence to light. Future studies, with tumor-specific tracers, should, therefore, strive to add the assessment of postoperative pathological NIRF images to the study designs.

## 5. Conclusions

Surgical resection is the cornerstone of treatment in GIST, and accurate tumor identification is essential for achieving complete and minimally invasive resections. With the rise in laparoscopic- and robotic-assisted surgery, the importance of optimal intraoperative tumor identification and demarcation is increasing. In this regard, NIRF imaging is hypothesized to be able to assist surgeons. Nevertheless, the current study shows that the intraoperative identification of GISTs with NIRF imaging, after intraoperatively administering ICG, is not feasible. Subsequent studies should aim to develop and explore the possibilities of fluorescence tracers that bind GIST-specific biomarkers.

## Figures and Tables

**Table 1:** Demographics, tumor characteristics, and treatments per patient. * Regorafenib after earlier courses of imatinib and sunitinib.

Patient Demographics, Tumor Characteristics and Treatment								
Patient	Center	Age	Sex	Presentation	Location	Size (mm)	Growth	Number of Lesions
1	LUMC	52	Female	Primary	Stomach	44	Exophytic	1
2	EMC	60	Male	Primary	Stomach	75	Intraluminal	1
3	LUMC	57	Male	Primary	Ileum	52	Exophytic	1
4	LUMC	66	Male	Primary	Stomach	90	Exophytic	1
5	LUMC	53	Male	Recurrence	Ileum	58	Exophytic	2
6	LUMC	61	Male	Primary	Duodenum	36	Exophytic	1
7	LUMC	80	Female	Primary	Stomach	48	Intraluminal	1
8	EMC	45	Male	Primary	Stomach	45	Exophytic	1
9	LUMC	46	Male	Primary	Stomach	25	Submucosal	1
10	EMC	56	Female	Primary	Stomach	150	Exophytic	1
**Patient**	**Mutation**			**Mitoses**	**Neoadjuvant Treatment**		**Surgery**	**Viable Cells Remaining**
1	PDGFRA exon 18			2	-		Laparoscopy	-
2	KIT exon 11			2	Imatinib		Laparotomy	100%
3	KIT exon 11			8	Imatinib		Laparotomy	75%
4	KIT exon 11			33	-		Laparoscopy	-
5	KIT exon 11			47	Regorafenib *		Laparotomy	100%
6	KIT exon 11			-	Imatinib		Laparotomy	50%
7	KIT exon 11			3	-		Laparoscopy	-
8	Not specified			7	-		Laparotomy	-
9	KIT exon 11			2	-		Laparoscopy	-
10	KIT exon 11			-	Imatinib		Laparotomy	0%

## Data Availability

The data presented in this study are available on request from the corresponding author.
